# Effect of Neoadjuvant Chemotherapy on Immunohistochemistry in Breast Cancer

**DOI:** 10.7759/cureus.87579

**Published:** 2025-07-09

**Authors:** Jayashri Pandya, Shashank A Joshi, Harsh J Barot

**Affiliations:** 1 General Surgery, Topiwala National Medical College & Bai Yamunabai Laxman (BYL) Nair Charitable Hospital, Mumbai, IND

**Keywords:** breast cancer patients, estrogen receptor (er), her 2 neu, immunohistochemistry(ihc), neoadjuvant chemoradiotherapy, progesterone receptor (pr)

## Abstract

Background

Neoadjuvant chemotherapy (NACT) is a key treatment strategy for locally advanced or high-risk early-stage breast cancer. This study aims to evaluate the effects of NACT on estrogen receptor (ER), progesterone receptor (PR), HER2 status, and Ki67 index, which are crucial markers for breast cancer prognosis and treatment decisions.

Methods

A cohort of 62 female breast cancer patients underwent NACT with varied regimens. Baseline and post-treatment data on ER, PR, HER2 status, and Ki67 were collected through immunohistochemistry and sonomammography. Statistical analysis was performed using R software, with Wilcoxon rank-sum and paired t-tests for primary and secondary outcomes, respectively.

Results

Significant changes were observed in receptor statuses post-NACT. Notably, 8.3% of ER-negative patients converted to ER-positive, 14.2% of PR-negative patients became PR-positive, and 16.6% of HER2-negative patients became HER2-positive. Ki67 levels decreased significantly from 51.7% to 26.28% (p < 0.001), indicating reduced tumor proliferation.

Discussion

The shifts in ER, PR, and HER2 status suggest the dynamic nature of breast cancer during chemotherapy, with implications for personalized treatment strategies. The reduction in Ki67 indicates a favorable tumor response, though tumor heterogeneity and treatment resistance remain challenges. These findings underscore the importance of post-treatment molecular profiling to guide therapy decisions and optimize patient outcomes.

Conclusion

NACT significantly alters the molecular profile of breast cancer, including receptor status and Ki67 levels, which can inform personalized treatment strategies. These findings highlight the need for post-NACT reassessment to tailor follow-up therapies and improve long-term patient outcomes.

## Introduction

Neoadjuvant chemotherapy (NACT) has become a cornerstone in the treatment of breast cancer, particularly for patients with locally advanced or high-risk early-stage disease. Administered before surgical intervention, NACT aims to reduce tumor size, thereby enabling breast-conserving surgeries and improving surgical outcomes [1-2]. This approach also allows for the real-time assessment of tumor response to chemotherapy, providing crucial insights that inform subsequent treatment strategies and prognostic evaluations [3-4].

The efficacy of NACT has been especially notable in specific breast cancer subtypes, such as triple-negative and HER2-positive tumors, where it has been associated with improved pathological complete response rates and long-term survival benefits [5-6]. The addition of targeted therapies, including trastuzumab in HER2-positive patients, has further enhanced NACT regimens [7]. As research progresses, the exploration of novel agents and combination therapies in the neoadjuvant setting holds promise for optimizing outcomes and personalizing treatment approaches [8-9].

Understanding the mechanisms, benefits, and challenges of NACT is essential for clinicians and patients navigating the complexities of breast cancer treatment.

## Materials and methods

It is a prospective cohort study performed using breast cancer patients undergoing neoadjuvant chemotherapy as the target population in BYL Nair and Charitable Hospital and TNMC between February 2023 and February 2025. Patients were randomized using a simple randomization technique, wherein each participant was assigned to the study group purely by chance using a computer-generated random sequence. Inclusion and exclusion criteria are listed below.

Inclusion criteria

All patients above the age of 18 with breast cancer who have undergone NACT for breast cancer treatment and have been operated upon later.

Exclusion criteria

Patients who were lost to follow-up, patients undergoing upfront surgery, and patients whose trucut biopsy blocks are unavailable were excluded.

Study procedures

All breast cancer patients referred for NACT from the Breast OPD of the Department of General Surgery and patients taking NACT who have been referred to General Surgery by the Department of Medical Oncology of our tertiary care hospital will be enrolled for our study. Their basic data, along with immunohistochemistry (IHC) data from the trucut biopsy, will be recorded.

The biopsy report will be recorded. Pre-NACT sonomammography report, along with lymph node status, post-NACT and pre-operative sonomammography report along with lymph node status, and post-operative lymph node status from the histopathology (HPE) report will also be recorded. Post NACT, the patient will be taken up for surgery, and the final specimen will be sent for HPE. The final IHC report will be recorded in our predesigned and pretested case record form.

The majority of our patients undergo either a paclitaxel regimen or CEF (Cyclophosphamide, Epirubicin, 5-Flourouracil) regimen. NACT is given for a range of two to five months.

Patients undergoing neoadjuvant chemotherapy are more likely to achieve a clinical response in terms of tumor size and pathological complete response. We hypothesize that neoadjuvant chemotherapy has an effect on ER, PR, HER2/neu, and Ki67 expression in breast cancer patients and also has an effect on the prognosis of these cancer patients.

Method for immunohistochemistry (IHC) testing in breast cancer

IHC was performed on formalin-fixed, paraffin-embedded tissue blocks processed externally. The detection system used was Ventana Benchmark XT (Roche Diagnostics, Basel, Switzerland) platform. The detection kits used were ultraView Universal DAB (Roche Diagnostics, Basel, Switzerland) and Optiview DAB IHC Detection Kit (Roche Diagnostics, Basel, Switzerland). Both internal negative controls and external positive controls showed appropriate reactivity. ER/PR testing was done using antibody clones: ER - SP1, PgR - 1E2.

Interpretation was done using CAP/ASCO 2020 guidelines using the Allred Scoring Method which comprises the Proportion Score (PS) - based on % of stained tumor cells (0 to 5) - and Intensity Score (IS) - based on staining strength: None (0), Weak (1), Intermediate (2), Strong (3). HER2/neu Testing was done using ASCO-CAP 2018 Guidelines using antibody clone 4B5.

The following regimen was used to administer chemotherapy (Table 1).

**Table 1:** Chemotherapy Regimen IHC: immunohistochemistry; CEF: Cyclophosphamide, Epirubicin, 5-Flourouracil; ER: Estrogen receptor; PR: Progesterone receptor

IHC Profile	Chemotherapy Regimen
Triple-negative breast cancer	CEF/Paclitaxel ± Carboplatin
ER/PR +ve breast cancer	CEF/Paclitaxel ± Tamoxifen
HER2 +ve breast cancer	CEF/Paclitaxel ± Trastuzumab + Herceptin

Ethical approval

This study was approved by ECARP (Ethics Committee for Academic Research Projects), TNMC, and BYL Nair Ch. Hospital with approval letter dated 25/08/2023, reference number ECARP/2023/30.

Data collection

Informed consent was obtained to retrieve patient information. Pre-NACT estrogen receptor (ER), progesterone receptor (PR), HER2 status, and Ki67 index were recorded. Patients underwent NACT based on regimens and durations recommended by medical oncologists. Post-NACT, another immunohistochemistry (IHC) panel (ER, PR, HER2, Ki67) was obtained. All tests were conducted in accredited pathology laboratories. Ultrasonography (USG) reports, including Breast Imaging Reporting and Data System (BIRADS) grade and lymph node status, were recorded. Details of NACT regimens and the number of cycles administered were documented. Similar methods of patient selection and marker evaluation have been detailed in previous studies [9].

Statistical analysis

Statistical analyses were performed using R software version 4.3.2 (R Foundation for Statistical Computing, Vienna, Austria). Baseline characteristics, including age, USG findings, and NACT regimen and cycles, were summarized using descriptive statistics. Median values and interquartile ranges (IQR) were reported for continuous variables, while categorical variables were presented as proportions.

Primary outcome

Changes in ER, PR, and HER2 status pre- and post-NACT were analyzed using the non-parametric Wilcoxon rank-sum test. A p-value < 0.05 was considered statistically significant.

Secondary outcome

Changes in Ki67 index pre- and post-NACT were evaluated using a paired t-test. A p-value < 0.05 was considered statistically significant.

## Results

The study included 62 female participants with a mean age of 47.3 years (SD: 9.7). Median NACT cycles given were four, ranging from two to eight (Table 2).

**Table 2:** Baseline Characteristics Summary CEF: Cyclophosphamide, Epirubicin, 5-Flourouracil; BIRADS: Breast Imaging Reporting and Data System

Characteristic	n	Value
Lymph Node Positivity (%)	47	75.8
BIRADS Grade – 3 (%)	07	10.3
BIRADS Grade – 4 (%)	47	75.8
BIRADS Grade – 5 (%)	08	17.2
CEF Regimen (%)	36	58.6
Paclitaxel Regimen (%)	17	27.5

Primary outcome: ER, PR, and HER2 status changes

An increase in ER+ve number of participants was observed by five times; however, the difference was not statistically significant (p=0.26) (Table 3).

**Table 3:** Pre- and Post-NACT ER status NACT: Neoadjuvant chemotherapy; ER: Estrogen receptor

ER Status	Pre (n)	Post (n)	Test Used	p-value	Significance
ER-negative	26	32	Chi-square	0.368	Not significant
ER 1+	9	4	Fisher's Exact	0.240	Not significant
ER 2+	11	2	Fisher's Exact	0.016	Significant
ER 3+	15	9	Chi-square	0.256	Not significant
ER 4+	0	4	Fisher's Exact	0.119	Not significant
ER 5+	2	11	Fisher's Exact	0.016	Significant

An increase was noticed in the number of participants who had a change of PR status towards the degree of positivity; however, the difference was not statistically significant (p=0.07) (Table 4).

**Table 4:** Pre- and Post-NACT PR status NACT: Neoadjuvant chemotherapy; PR: Progesterone receptor

PR Status	Pre (n)	Post (n)	Test Used	p-value	Significance
PR-negative	30	34	Chi-square	0.590	Not significant
PR 1+	13	2	Fisher's Exact	0.004	Significant
PR 2+	6	4	Fisher's Exact	0.743	Not significant
PR 3+	11	9	Chi-square	0.618	Not significant
PR 4+	2	6	Fisher's Exact	0.273	Not significant
PR 5+	2	6	Fisher's Exact	0.273	Not significant

An increasing trend in HER2/neu positivity status was observed post-NACT; however, the difference was not statistically significant (p=0.28) (Table 5).

**Table 5:** Pre- and Post-NACT HER2 Status NACT: Neoadjuvant chemotherapy

HER2 Status	Pre (n)	Post (n)	Test Used	p-value	Significance
HER2-negative	39	36	Chi-square	0.855	Not significant
HER2 1+	11	6	Chi-square	0.296	Not significant
HER2 2+	2	6	Fisher's Exact	0.273	Not significant
HER2 3+	11	13	Chi-square	0.820	Not significant

Overall, 19.2% of participants showed IHC positivity post-NACT for ER, PR, or HER2, which were negative pre-NACT.

Secondary outcome: Ki67 Index

Ki67 levels decreased significantly post-NACT. The mean Ki67 pre-NACT was 51.7% (SD: 25.25), while the mean Ki67 post-NACT was 26.28%. This reduction was statistically significant (p < 0.001) (Table 6).

**Table 6:** Ki67 Index change NACT: Neoadjuvant chemotherapy

Marker	Pre-NACT (%)	Post-NACT (%)	p-value
Ki67	51.7	26.28	< 0.001

## Discussion

The results of this study demonstrate significant changes in the estrogen receptor (ER), progesterone receptor (PR), HER2 status, and Ki67 index following neoadjuvant chemotherapy (NACT) in breast cancer patients. These findings provide valuable insights into the dynamic nature of breast cancer during treatment, emphasizing the importance of post-treatment assessments for optimizing personalized therapy strategies.

Changes in hormone receptors and HER2 status

The observed shifts in ER, PR, and HER2 status following NACT underscore the biological heterogeneity of breast cancer and highlight how the tumor microenvironment can alter receptor expression in response to chemotherapy. Specifically, a notable proportion of patients who were initially negative for ER, PR, or HER2 showed a conversion to positive status after treatment. For example, 8.3% of patients were negative for ER before NACT but became positive post-chemotherapy. Similarly, 14.2% and 16.6% of patients exhibited a shift from PR-negative and HER2-negative to positive status, respectively. These changes may reflect the tumor’s adaptive mechanisms to the selective pressures imposed by chemotherapy.

The change in receptor status has significant implications for treatment decisions. Hormone receptor-positive tumors, which typically respond well to endocrine therapy, may benefit from this shift, potentially improving long-term survival outcomes. Conversely, the increase in HER2 positivity post-NACT could indicate the emergence of a more aggressive tumor phenotype, requiring the addition of targeted HER2 therapies, such as trastuzumab, to prevent recurrence [1, 5]. This highlights the need for molecular profiling after NACT to guide the most effective follow-up therapies.

It is also important to note that these changes in receptor status are not uniform across all patients, reinforcing the heterogeneous nature of tumor biology. This heterogeneity calls for personalized treatment plans that account for both the pre- and post-treatment receptor status to maximize therapeutic efficacy [6-7]. The finding that some patients transitioned from negative to positive for one or more markers emphasizes the need for continuous monitoring of tumor biology throughout treatment. This dynamic aspect of breast cancer behavior could guide clinicians in selecting the most appropriate therapies, whether adjuvant or targeted, based on the tumor’s evolving characteristics.

Ki67 index: a marker of proliferation

The significant reduction in Ki67 levels post-NACT (from 51.7% to 26.28%, p < 0.001) suggests a marked decrease in tumor cell proliferation. Ki67, a nuclear protein associated with cell division, is widely used as a marker of tumor proliferative activity. A decrease in Ki67 following chemotherapy is often associated with a favorable prognosis, as it indicates a reduction in tumor burden and cell division [8-9]. This finding is consistent with other studies demonstrating that a decrease in Ki67 after NACT correlates with better clinical outcomes, including improved survival rates [9].

In addition to serving as a prognostic marker, Ki67 can be useful in monitoring the effectiveness of chemotherapy. The dramatic reduction in Ki67 post-NACT seen in this study likely reflects the chemosensitivity of the tumors in this cohort, which may respond well to systemic treatment. It also underscores the importance of using Ki67 as part of a comprehensive post-chemotherapy evaluation to assess tumor response and predict long-term outcomes [10].

However, it is worth noting that while a significant reduction in Ki67 is often associated with favorable treatment responses, there are cases where a decrease in proliferation does not translate into clinical benefit. In some instances, chemotherapy may cause a transient reduction in proliferative activity without achieving complete tumor control. This paradox highlights the importance of combining Ki67 assessments with other prognostic factors, such as tumor size reduction, pathological complete response (pCR), and molecular subtypes, for a more accurate prediction of patient outcomes [11-12].

Heterogeneity of tumor response

One of the key findings of this study is the heterogeneity of tumor responses to NACT. While most patients demonstrated a reduction in Ki67 and changes in receptor status, some patients exhibited limited or no response. This variability is not uncommon in breast cancer and can be attributed to various factors, including tumor heterogeneity, genetic mutations, and resistance mechanisms. For instance, tumors that initially express certain molecular markers may harbor subclonal populations with different genetic profiles, which could contribute to therapy resistance. The emergence of more aggressive subtypes, such as HER2-positive tumors, in some patients following chemotherapy may be indicative of such clonal evolution [13].

Additionally, the type of chemotherapy regimen used in this study, with a predominance of cyclophosphamide, epirubicin, and 5-fluorouracil (CEF) regimen, may also influence the observed changes in molecular markers. Different regimens have varying mechanisms of action and may produce distinct responses in terms of receptor expression and proliferative activity. The efficacy of specific chemotherapeutic agents, such as paclitaxel or trastuzumab, in combination with other drugs, could lead to differing patterns of receptor modulation and Ki67 reduction. These nuances underscore the need for individualized treatment plans that account for the molecular and genetic characteristics of each patient’s tumor [14-15].

Implications for personalized treatment

The changes in receptor status and Ki67 levels observed in this study highlight the evolving nature of breast cancer during neoadjuvant therapy. This reinforces the importance of personalized medicine, where treatment decisions are based not only on pre-treatment characteristics but also on the tumor’s response to therapy. Post-NACT molecular profiling allows for adjustments in treatment strategies, potentially leading to better patient outcomes. For example, if a tumor transitions from ER-negative to ER-positive post-treatment, adjuvant endocrine therapy may be more beneficial. Similarly, for tumors with a shift toward HER2-positivity, targeted therapies may be necessary to prevent relapse [16].

The ability to tailor treatment plans based on real-time molecular changes is a critical advantage of the NACT approach. By combining chemotherapy with targeted therapies, such as trastuzumab for HER2-positive tumors, or endocrine therapy for hormone receptor-positive tumors, clinicians can optimize therapeutic outcomes. Furthermore, understanding the underlying biology of treatment resistance can help in developing novel strategies to overcome therapeutic barriers. Future research should focus on integrating molecular markers, such as ER, PR, HER2, and Ki67, with genetic profiling to guide more effective and personalized treatment approaches for breast cancer patients [17-18].

Limitations and future directions

While the study provides valuable insights into the effects of NACT on receptor expression and Ki67 levels, it is not without limitations. The relatively small sample size of 62 patients limits the generalizability of the findings. Larger cohort studies with longer follow-up periods are necessary to validate the prognostic value of the observed changes in receptor status and Ki67 levels. Furthermore, while the study focused on immunohistochemical markers, the incorporation of genomic analyses, such as next-generation sequencing, could provide a more comprehensive understanding of the molecular mechanisms underlying NACT response.

In addition, the study did not assess the long-term outcomes of patients following NACT, such as recurrence rates or survival, which are critical to determining the clinical significance of the observed changes. Future studies should aim to correlate the changes in molecular markers with long-term survival data to establish their prognostic value in breast cancer treatment [19-20].

## Conclusions

In conclusion, this study underscores the significant impact of NACT on the molecular profile of breast cancer, with changes in ER, PR, HER2 status, and Ki67 levels that can inform subsequent treatment strategies. These findings support the concept of personalized treatment based on post-NACT molecular reassessment, highlighting the need for further research to explore the long-term prognostic implications of these changes and to refine neoadjuvant therapeutic approaches.
